# Role of the Senescence-Associated Factor Dipeptidyl Peptidase 4 in the Pathogenesis of SARS-CoV-2 Infection

**DOI:** 10.14336/AD.2023.0812

**Published:** 2024-05-07

**Authors:** Stefanie Deinhardt-Emmer, Sharvari Deshpande, Koji Kitazawa, Allison B. Herman, Joanna Bons, Jacob P. Rose, Prasanna Ashok Kumar, Carlos Anerillas, Francesco Neri, Serban Ciotlos, Kevin Perez, Nilay Köse-Vogel, Antje Häder, Kotb Abdelmohsen, Bettina Löffler, Myriam Gorospe, Pierre-Yves Desprez, Simon Melov, David Furman, Birgit Schilling, Judith Campisi

**Affiliations:** ^1^Buck Institute for Research on Aging, Novato, CA 94945, USA.; ^2^Institute of Medical Microbiology, Jena University Hospital, Germany.; ^3^Laboratory of Genetics and Genomics, National Institute on Aging, Intramural Research Program, National Institutes of Health, Baltimore, MD 21224, USA.; ^4^Stanford 1000 Immunomes Project, Stanford University School of Medicine, Stanford, CA 94305, USA.; ^5^Buck Artificial Intelligence Platform, Buck Institute for Research on Aging, Novato, CA 94945, USA.

**Keywords:** COVID-19, cellular senescence, DDP4, infection

## Abstract

During cellular senescence, persistent growth arrest and changes in protein expression programs are accompanied by a senescence-associated secretory phenotype (SASP). In this study, we detected the upregulation of the SASP-related protein dipeptidyl peptidase 4 (DDP4) in human primary lung cells rendered senescent by exposure to ionizing radiation. DPP4 is an exopeptidase that plays a crucial role in the cleavage of various proteins, resulting in the loss of N-terminal dipeptides and proinflammatory effects. Interestingly, our data revealed an association between severe coronavirus disease 2019 (COVID-19) and DDP4, namely that DPP4 levels increased in the plasma of patients with COVID-19 and were correlated with age and disease progression. Although we could not determine the direct effect of DDP4 on viral replication, mechanistic studies in cell culture revealed a negative impact on the expression of the tight junction protein zonula occludens-1 (ZO-1), which contributes to epithelial barrier function. Mass spectrometry analysis indicated that DPP4 overexpressing cells exhibited a decrease in ZO-1 and increased expression of pro-inflammatory cytokines and chemokines. By investigating the effect of DPP4 on the barrier function of human primary cells, we detected an increase in ZO-1 using DPP4 inhibitors. These results provide an important contribution to our understanding of DPP4 in the context of senescence, suggesting that DPP4 plays a major role as part of the SASP. Our results provide evidence that cellular senescence, a hallmark of aging, has an important impact on respiratory infections.

## INTRODUCTION

Aging is characterized by several hallmarks that contribute to a gradual reduction of physiological integrity [1]. One such characteristic feature is cellular senescence, which represents a state of stable growth arrest combined with a senescence-associated secretory phenotype (SASP). During early life, the SASP can promote immune function and wound healing, and it can prevent tumorigenic transformation [2]. However, the SASP can exert harmful effects in older individuals, promoting carcinogenesis, and exacerbating infections and immune responses [3, 4].

The SASP comprises various molecules, including growth factors, cytokines, and active inflammatory substances [5, 6]. Upregulation of dipeptidyl peptidase 4 (DPP4) has been described in multiple tissues where senescence is detectable [7]. DPP4, also known as CD26, is a serine exopeptidase with high catalytic activity. Enzymatic activity leads to the cleavage of X-proline or X-arginine amino acids from the N-terminal side. DPP4 protein is present both in transmembrane-bound form in various cell types and in soluble form (sDPP4). Multiple functions have been described for DPP4, including an exopeptidase function with crucial roles in the cleavage of various proteins, resulting in the loss of N-terminal dipeptides with pro-inflammatory effects [8].

In this study, we aimed to investigate the expression of DPP4 in senescent lung cells and characterize its influence on viral infections. Patients with pre-existing conditions are particularly affected by severe infection courses, with research into the novel severe acute respiratory syndrome coronavirus 2 (SARS-CoV-2) showing that aging plays a significant role in increasing mortality [9]. In particular, patients over 65 years of age suffer more frequently from severe coronavirus disease 2019 (COVID-19), with high mortality rates [10].

Here, we show that DPP4, also described as a receptor for the host-cell binding of SARS-CoV-2, is upregulated in various types of senescent lung cells. We also show a relationship between age, DPP4, and the severity of COVID-19. The spike (S) glycoprotein is responsible for the entry of SARS-CoV-2 and is composed of two parts, S1 and S2 [11]. The receptor-binding protein (RBD) of the S protein interacts with several host cell proteins, of which angiotensin-converting enzyme 2 (ACE 2) has the highest binding affinity for the virus [12]. However, other receptors have also been characterized as targets of viral entry [12]. Among them is DDP4, which was previously identified as a co-receptor during the MERS outbreak in 2012 [13], and now also exhibits a potential binding capacity to SARS-CoV-2 [14, 15].

DPP4 is a highly active proteolytic protein involved in the cleavage of various substrates, such as glucagon-like-peptide [16]. The overall relevance of DPP4 as a protease for protein cleavage has been reported for several substrates [8]. However, the potential role of DPP4 in the cleavage of the spike protein of SARS-CoV-2 is still unclear. Vankadari et al. [17] proposed that the S protein interacts with DPP4, and Li et al. [15] described the MERS receptor as a potential co-receptor for SARS-CoV-2. Recently published data have indicated various viral cleavage sites in the spike protein [18].

While our study was unable to identify a direct effect on viral replication mediated by DPP4, mass spectrometry analysis revealed the effects of DPP4 knockout and overexpression in lung cells on inflammatory parameters and tight-junction proteins. Through functional studies, we demonstrate that DPP4 negatively affects barrier function by reducing the levels of the protein zonula occludens 1 (ZO-1, encoded by the *TJP1* gene). Thus, our study contributes to a deeper understanding of the association between cellular senescence and respiratory infection.

## MATERIALS AND METHODS

### Cell cultures

The following human primary lung cells were used for viral infections: human fetal lung fibroblasts (IMR-90), human SAECs, and human lung microvascular endothelial cells (HMVEC-L). Human IMR-90 cells were obtained from the Coriell Institute for Medical Research (#I90, Camden, NJ, USA) and cultured in Dulbecco’s modified Eagle medium (DMEM) (#11960044, Gibco, Waltham, MA, USA) supplemented with 10% fetal bovine serum (FBS) (#26140079, Gibco, Waltham, MA, USA) and penicillin/streptomycin (#15140122, Gibco, Waltham, MA, USA). Quiescence was achieved by replacing the culture medium with 0.2% FBS for 72 h, then with serum-free media for 24 h before collection [5, 6]. The cells were cultured in 3% O_2_ and 5% CO_2_ at 37 °C. Human SAECs were obtained from Lonza (#CC-2547, Bend, OR, USA) and cultured in SAGMTM small airway epithelial cell growth medium (#CC-3118, Lonza, Bend, OR, USA) in 20% O_2_ and 5% CO_2_ at 37 ºC. Human HMVEC-L were obtained from Lonza (#CC-2527, Bend, OR, USA) and cultured in EGMTM -2 MV microvascular endothelial cell growth medium-2 (#CC-3202, Lonza, Bend, OR, USA). The cells were maintained in 20% O_2_ and 5% CO_2_ at 37 °C.

Human kidney HEK293-FT cells were obtained from ThermoFisher (#R70007, Waktham, MA, USA) and cultured in DMEM (#11960044, Gibco, Waltham, MA, USA) supplemented with 10% FBS and penicillin/streptomycin (#15140122, Gibco Waltham, MA, USA). The cells were incubated in 10% O_2_ and 5% CO_2_ at 37 °C. The human lung cancer cell line Calu-3, which was a gift from the Robert Koch Institute (Berlin, Germany), was cultured in DMEM (#11960044, Gibco, Waltham, MA, USA) supplemented with 20% FBS and penicillin/streptomycin (#15140122, Gibco, Waltham, MA, USA), then incubated in 3% O_2_ and 5% CO_2_ at 37 °C. Vero-76 cells were used for viral propagation. These cells were cultured in Eagle´s Minimum Essential Medium (EMEM) (#M4655, Sigma Aldrich, St. Louis, USA) supplemented with HEPES (#7365-45-9 Sigma Aldrich, St. Louis, USA) buffer and 5 mM L-glutamine (#G7513, Sigma Aldrich, St. Louis, USA) in 3% O_2_ and 5% CO_2_ at 37 ºC.

Primary CECs were obtained from human donor corneas (CorneaGen, Seattle Eye Bank, WA) and cultured at 37 °C under 95% humidity and 5% CO_2_, as previously described [19]. CECs were cultured in Dulbecco’s modified Eagle’s medium and Ham’s F-12 medium (#51445C, DMEM/F12, 1:1 mixture, Sigma Aldrich, St. Louis, USA), B-27TM supplement (2%) (#17504044, Thermo Fisher Scientific, Waltham, MA, USA), Rho-kinase inhibitor Y-27632 (10 µM) (#S1049, Selleck Chemicals, Houston, TX, USA), keratinocyte growth factor (10 ng/ml) (#S0015, Thermo Fisher Scientific, Waltham, MA, USA), and penicillin-streptomycin (50 IU/ml).

Human diploid WI-38 fibroblasts (Coriell Institute) and IMR-90 fibroblasts (ATCC) were cultured in DMEM (Gibco) supplemented with 10% heat-inactivated FBS (Gibco) in a 5% CO_2_ incubator. Cells were maintained at low population doubling levels (PDL) for the experiments in this study.

### SARS-CoV-2 propagation and infection

Viral infection was induced using a SARS-CoV-2 strain isolated from a respiratory specimen of the patient (SARS-CoV-2/hu/Germany/Jena-vi005588/2020 (5588, GenBank: MW633324), SARS-CoV-2/hu/Germany/ Jena-vi043115/2021, SARS-CoV-2/hu/Germany/Jena-0114749/2021 (GenBank: ON650061.1)), under ethics approval of the Jena University Hospital, no. 2018-1263. Propagation of the virus strains was performed using Vero-76 cells, as described previously [20]. IMR-90 cells were infected with this SARS-CoV-2 strain at a multiplicity of infection of 1 for 120 min in DMEM supplemented with 10% FBS. The infection medium was then removed, and fresh medium was added at the indicated time points.

### SARS-CoV-2 pseudotyped lentiviral infection

To determine the ability of SARS-CoV-2 to attach and enter the cells via DPP4, we used IMR-90 cells overexpressing DPP4. After treatment with Poly-L-Lysine (Sigma Aldrich, #25988-63-0, MA, USA), cells were seeded for 24 h, followed by infection with SARS-CoV-2 pseudotyped lentiviral particles produced as we previously published [21]. Briefly, the pseudotyped virus was created using a luciferase-expressing SARS-CoV-2 spike plasmid. Pseudovirus was removed after an 1 h incubation. As a positive control, we used vesicular stomatitis virus glycoprotein G (VSV-G). After 48 h, luciferase assay was performed (Promega, #E1501, WI, USA) using a TECAN reader to detect luminescence. A luminescence signal was detectable only if the pseudotyped virus was able to enter the cells, indicating a binding between receptor and spike proteins.

### Animal experiments

Young adult C57BL/6 mice (12-13 weeks old) were purchased from Charles River Laboratories and acclimatized to the housing conditions of the Buck Institute for Research on Aging for two weeks. Old adult C57BL/6 mice (more than 90 weeks old) were aged in-house under standard Biosafety Level 2 conditions at the Buck Institute (IACUC protocol #A10250). The mice were kept under 12:12 light:dark conditions with *ad libitum* access to food and water. At the indicated time points, the mice were immediately euthanized using CO_2_. For BAL, the thorax was opened, and the trachea was exposed. The trachea was punctured with a needle and 500 µL of pre-warmed sterile Dulbecco´s phosphate buffered saline (DPBS) (#D8537, Sigma Aldrich, St. Louis, USA) solution were applied. After the resumption of DPBS, the fluid was immediately frozen in liquid nitrogen.

### Senescence induction

Senescence was induced by ionizing irradiation (IR) at a dose of 15 Gy [5]. Quiescent (non-senescent) controls were mock-irradiated by placing them in an X-ray machine for an equivalent amount of time. Subsequently, the media were replaced with fresh complete media and changed every two days. On day 7, cells were cultured in low-serum media (0.2% FBS for 72 h), then in serum-free media for 24 h before collecting the CM [5].

The primary CECs were seeded in six-well plates and treated with the chemotherapeutic agent doxorubicin (DOXO) (#25316-40-9, 250 nM, Selleck Chemicals, Houston, TX, USA) or vehicle (DMSO, # 472301, Sigma Aldrich, St. Louis, USA) as a control for 24 h to induce cellular senescence from disruption of topoisomerase-II-mediated DNA repair. The cells were then cultured for 10 d to allow development of senescent features [22, 23].

For human diploid WI-38 fibroblasts (Coriell Institute), cellular senescence was triggered by treatment with etoposide (#S1225, 50 μM, Selleck Chemicals, Houston, TX, USA) for 8-10 days, or by maintaining cells in culture until replicative exhaustion (beyond PDL 50). BrdU incorporation and senescence-associated β-galactosidase assays were performed according to the manufacturer’s protocol (#6813, Cell Signaling Technology, MA, USA).

### Barrier function assay

DOXO-treated CECs (senescent cells; SnC) and DMSO-treated CECs (non-senescent cells; NS) were seeded at a cell density of 100,000 cells in 24-well transwell plates (#CLS3378, Corning, 24-mm Transwell with 0.4-μm Pore Polyester Membrane Insert, Sigma Aldrich, St. Louis, USA) at a SnC to NS ratio of 75:25 or at 100% NS. To measure the barrier function of CECs, the cells were tested for TEER between the upper and lower chambers of permeable cell culture inserts using a volt-ohm meter (EVOM, World Precision Instruments, Sarasota, FL), as previously reported [24].

The TEER was measured and calculated by multiplying the measured resistance (ohms) by the culture area of the filter using the volt-ohm meter. The background resistance owing to the filter alone was subtracted from each measurement. TEER was measured twice, and the average value was used for the analysis. The cells were treated for 24 h with the DPP4 inhibitor (50 µM), recombinant DPP4 (50 µg/ml), or vehicle (DMSO).

### Conditioned media

SnC and NS were cultured in complete CEC media, washed three times with PBS, and placed in DMEM/F12 (serum- and supplement-free media) (Thermo Fisher Scientific) for 24 h. Subsequently, the CM was collected. A total of 200,000 cell equivalents per milliliter of CM were used for the barrier function assay.

### RNA extraction and real-time PCR

RNA isolation from cells in culture was performed using Meridian Biosciences (#BIO-52073, Cincinnati, OH, USA) and from animal tissue using the Zymogen Direct-zol RNA Miniprep kit (#R2051, Modena, Italy), according to the manufacturer’s instructions. Approximately 50 ng/ml of RNA was used to synthesize cDNA. mRNA expression analysis was performed by LightCycler 480 qPCR using a universal probe library (F. Hoffmann-LaRoche, CA, USA), and mRNA levels were normalized to actin levels.

### Single-cell sequencing

IMR-90 cells were induced to senescence using doxorubicin at a final concentration of 250 nM (from a stock of 250 µM in DMSO) and after treatment for 24 h. Cell growth arrest was confirmed by cell counting and RNA was harvested 10 days after treatment. Quiescent and proliferating cells were cultured in 0.2% and 10% FBS, respectively, and RNA was harvested after 3-4 days. The samples were processed using the Chromium Single Cell A Chip Kit and Chromium Controller (10X Genomics). Quality control was performed using a Tapestation 4200 instrument (Agilent Technologies). Libraries were sequenced in one lane of NovaSeqS4 by the University of California Davis Genomics Core. Reads were mapped to the human genome using Cell Ranger (10X Genomics) and GRCh38 genome reference. Cells were not analyzed if they expressed <200 unique genes, >4,000 unique genes, or >15% of mitochondrial genes. Genes not detected in any cell were removed from subsequent analysis. After read count normalization, variable feature detection (nfeatures = 2,000), scaling, principal component analysis (PCA), and Uniform Manifold Approximation and Projection (UMAP) were computed as described in the Seurat package, utilizing 10 dimensions [25].

### ELISA for human and murine DPP4

To determine the DPP4 protein levels, 3x10^4^ cells were seeded and treated with IR or doxorubicin to induce senescence. The CM was collected 10 days post-treatment and centrifuged at 5,000 xg for 10 min. The supernatants were analyzed using the human DPPIV/CD26 ELISA kit (#EHDPP4, Thermo Fisher Scientific, Waltham, MA, USA) following the manufacturer’s instructions. For mouse experiments, BAL was performed to collect BAL fluid using endotoxin-free phosphate-buffered saline. BAL-DPP4 levels were measured using the DPPIV (DPP4/CD26) mouse ELISA kit (#EMDPP4, Thermo Fisher Scientific, Waltham, MA, USA) according to the manufacturer’s instructions.

### DPP4 activity assay

To determine the activity of DPP4 in culture and *in vivo*, we used the DPP4 activity assay kit (#MAK088-1KT, Sigma Aldrich, St. Louis, USA), where DPP4 cleaves the non-fluorescent substrate H-Gly-Pro-AMC into fluorescent 7-Amino-4-Methyl Coumarin. Following the manufacturer's instructions, 10 µl of the sample was added to 40 µl of assay buffer. For the master mix, we used 2 µl of substrate combined with 38 µl of assay buffer, incubated on a shaker and protected from light, followed by 5 min incubation at 37°C. We then measured the initial fluorescence intensity (FLU) (λex = 360 nm/λem = 460 nm) every 5 min to determine the activity level.

### Western blotting

Cells were lysed using a bead homogenizer (Qiagen, Redwood City, CA, USA) in 5% SDS and 10 mM Tris buffer, and the protein concentration was assessed using the PierceTM BCA Protein Assay kit (#23225, Thermo Fisher Scientific, Waltham, MA, USA) in a flat-bottom 96-well microplate, according to the manufacturer’s instructions. After 30 min of incubation, absorbance was measured at 563 nm using a plate reader. Protein separation was performed by electrophoresis and the proteins were transferred to polyvinylidene difluoride membranes. The membranes were washed with TBST + 5% BSA, incubated with the primary antibody overnight, washed with TBST, and incubated with an HRP-conjugated secondary antibody. The bands were visualized by chemiluminescence using SuperSignalTM West Pico PLUS Chemiluminescent Substrate (#34579, Thermo Fisher Scientific, Waltham, MA, USA). To quantify the amount of protein in Western blotting, we used ImageJ (imagej.nih.gov/ij/download) as described previously [26].

### Lentiviral constructs for DPP4 overexpression and knockout

To generate IMR-90 cells with DPP4 knockout or DPP4 overexpression, we prepared lentiviral particles. We used the following packaging plasmids for the lentiviral infection of HEK293-FT cells: pCMV-VSV-G (AddGene, plasmid #8454, Watertown, MA, USA) and psPAX2 (AddGene, plasmid #12260, Watertown, MA, USA). For DPP4 knockout, we used the DPP4 CRISP guide RNA1_pLentiCRISP (GenScript, U6021GC070-1, New Jersey, USA). For DPP4 overexpression, we used the pLEX307-DPP4-puro plasmid (AddGene, plasmid #158451, Watertown, MA, USA). To measure the concentration of lentiviral particles, we performed single-wash ELISA to determine p24 protein levels (Abcam, ab218268, Waltham, MA, USA).

### Immunofluorescence microscopy

Cells were fixed in ice-cold 100% methanol for 15 min at 4 °C, washed three times with PBS, and permeabilized with 0.1% Triton X-100 in PBS for 15 min at room temperature (RT). Cells were blocked in 3% BSA-PBS solution for 45 min at RT, followed by overnight incubation with DPP4 primary antibody (#MA2607, Thermo Fisher Scientific; Waltham, MA, USA) prepared at 1:20 dilution in 3% BSA-PBS. The next day, cells were washed in PBS and incubated at RT for 2 h with 488-conjugated IgG in BSA-PBS. Cells were then washed with PBS, stained with DAPI (1:2,000) for 5 min, and mounted for imaging.

To detect ZO-1, cells in culture plates were fixed with cold methanol at 4°C for 10 min. The fixative was aspirated, and cells were rinsed with PBS for 5 min and permeabilized with 0.05% Triton-X-100 at RT for 30 min. Cells were blocked with 10% goat serum at RT for 45 min, then incubated with a primary antibody against ZO-1 (Invitrogen, rabbit, 1:100 dilution) overnight at 4 °C. The next day, cells were washed with PBS and incubated with Alexa Fluor 488-conjugated anti-rabbit IgG (#A11070, Invitrogen, Thermo Fisher Scientific, Waltham, MA, USA) at a dilution of 1:1,000 for 1 h at RT. Finally, cells were viewed under a fluorescence microscope (Nikon Eclipse E800) and photographed.

Immunofluorescent staining of SARS-CoV-2 samples was performed in 24-well plates, and cells were cultured on coverslips. After infection, cells were fixed with 4% paraformaldehyde in PBS for at least 30 min at 37°C, permeabilized with PBS containing 0.1% Triton-X, and blocked with blocking solution (3% BSA in PBS) at RT for 30 min. The cover slips were incubated with different primary antibodies (SARS-CoV-2 spike antibody (1A9), GeneTex (#GTX632604, CA, USA), 1:500; CD26 Monoclonal Antibody (236.3), Invitrogen, #MA2607, 1:100, Thermo Fisher Scientific; Waltham, MA, USA) diluted in blocking solution 4°C overnight. Alexa Fluor® 488-conjugated donkey anti-rabbit and Cy-3 conjugated donkey anti-mouse (#715-165-150, Jackson Immunoresearch, Cambridgeshire, UK) solutions were applied at a dilution of 1:500. Finally, coverslips with stained cells were mounted with DAPI Fluoromount-G (#0100-20, Southern Biotech, Birmingham, AL, USA) on microscope slides and analyzed using an AxioObserver Z.1 microscope (Zeiss, Jena, Germany).

To measure the uptake of SARS-CoV-2 S (Spike) protein, 10^5^ cells were seeded per well in chamber slides (#177380, Thermo Fisher Scientific, Waltham, MA, USA) and treated with vehicle (DMSO) or etoposide for eight days to induce senescence. Cells were then cultured in the presence of 160 μg of His-tagged SARS-CoV-2 Spike Subunit 1 or 2 recombinant protein or no recombinant protein for 3 h. Cells were fixed with 4% formaldehyde, permeabilized with 0.2% Triton X-100, and blocked in 10% normal goat serum (#31872, Thermo Fisher Scientific, Waltham, MA, USA) for 1 h at 37°C. After blocking, cells were incubated with antibodies recognizing His-tag (Abcam) in 10% normal goat serum for 1 h at 37°C, then with Alexa Fluor 568 (#A-11011, Thermo Fisher Scientific, Waltham, MA, USA) in 10% normal goat serum for 30 min at 37°C. After washing with PBS, the nuclei were stained with DAPI (#D523, Dojindo Laboratories, Kumamoto, Japan) for 15 min at 25°C, and the cell preparation was covered with mounting medium. Signals were visualized using a Keyence microscope.

### SomaLogic protein assay

We performed 5,000 SomaLogic protein assays (SomaLogic, CO, USA) using plasma from patients with mild (n = 35), moderate (n = 10), and severe (n = 42) COVID-19 and from healthy volunteers (controls, n = 33). Differentially expressed genes were calculated using the R package DEseq2. P values were corrected for multiple comparisons using Bonferroni correction, an FDR threshold of < 0.05, and abs(logFC) > 1 for significance. For each gene, we investigated the association between mild, moderate, and severe COVID-19 cases and the controls.

### Proteomic analysis and sample concentration

15 ml of conditioned media from wild-type, DDP4 overexpressing, and DPP4 knockout IMR90 cells, in both senescent (10 Gy Xray) and quiescent conditions (N=24), was concentrated to 300 µl with 15 ml 3 kDa filters (Millipore Sigma, Burlington, MA). Protein concentration was determined using Bicinchoninic Acid (BCA) assay (Thermo Fisher Scientific, Waltham, MA, USA).

### Protein digestion and desalting

Aliquots of 200 µg protein lysates for each sample were reduced using 20 mM dithiothreitol in 50 mM triethylammonium bicarbonate buffer (#15715-58-9 TEAB, Sigma Aldrich, St. Louis, USA) at 50°C for 10 min, cooled to room temperature (RT) and held at RT for 10 min, and alkylated using 40 mM iodoacetamide in 50 mM TEAB at RT in the dark for 30 min. Samples were acidified with 12% phosphoric acid to obtain a final concentration of 1.2% phosphoric acid. S-Trap buffer consisting of 90% methanol in 100 mM TEAB at pH ~7.1, was added and samples were loaded onto the S-Trap mini spin columns. The entire sample volume was spun through the S-Trap mini spin columns at 4,000 x g and RT, binding the proteins to the mini spin columns. Subsequently, S-Trap mini spin columns were washed twice with S-Trap buffer at 4,000 x g at RT and placed into clean elution tubes. Samples were incubated for one hour at 47°C with sequencing grade trypsin (#VA9000, Promega, San Luis Obispo, CA, USA) dissolved in 50 mM TEAB at a 1:25 (w/w) enzyme:protein ratio. Afterwards, trypsin solution was added again at the same ratio, and proteins were digested overnight at 37°C.

Peptides were sequentially eluted from mini S-Trap spin columns with 50 mM TEAB, 0.5% formic acid (FA) in water, and 50% acetonitrile (ACN) in 0.5% FA. After centrifugal evaporation, samples were resuspended in 0.2% FA in water and desalted with Oasis 10 mg Sorbent Cartridges (#186000383, Waters, Milford, MA, USA). The desalted elutions were then subjected to an additional round of centrifugal evaporation and re-suspended in 0.2% FA in water at a final concentration of 1 µg/µl. Finally, indexed Retention Time standard peptides (iRT, Biognosys, Schlieren, Switzerland) were spiked in the samples according to manufacturer’s instructions [27].

### Mass Spectrometric Analysis

LC-MS/MS analyses were performed on a Dionex UltiMate 3000 system coupled to an Orbitrap Eclipse Tribrid mass spectrometer (both from Thermo Fisher Scientific, San Jose, CA). The solvent system consisted of 2% ACN, 0.1% FA in H2O (solvent A) and 98% ACN, 0.1% FA in H2O (solvent B). Proteolytic peptides (400 ng) were loaded onto an Acclaim PepMap 100 C18 trap column (0.1 x 20 mm, 5 µm particle size; Thermo Fisher Scientific) for 5 min at 5 µl/min with 100% solvent A. Peptides were eluted on an Acclaim PepMap 100 C18 analytical column (75 µm x 50 cm, 3 µm particle size; Thermo Fisher Scientific) at 300 nl/min using the following gradient of solvent B: 2% for 5 min, linear from 2% to 20% in 125 min, linear from 20% to 32% in 40 min, up to 80% in 1 min, 80% for 9 min, and down to 2% in 1 min. The column was equilibrated with 2% of solvent B for 29 min, with a total gradient length of 210 min.

All samples were acquired in data-independent acquisition (DIA) mode. Full MS spectra were collected at 120,000 resolutions (AGC target: 3e6 ions, maximum injection time: 60 ms, 350-1,650 m/z), and MS2 spectra at 30,000 resolutions (AGC target: 3e6 ions, maximum injection time: Auto, NCE: 27, fixed first mass 200 m/z). The isolation scheme consisted in 26 variable windows covering the 350-1,650 m/z range with an overlap of 1 m/z [28].

### DIA data processing and statistical analysis

DIA data were processed in Spectronaut (version 15.1.210713.50606) using directDIA. Data were searched against a human database containing all UniProt-SwissProt entries extracted on 07/31/2021 (20,386 entries). Trypsin/P was set as the digestion enzyme and two missed cleavages were allowed. Cysteine carbamidomethylation was set as a fixed modification while methionine oxidation and protein N-terminus acetylation were set as dynamic modifications. Data extraction parameters were set as dynamic and non-linear iRT calibration with precision iRT was selected. Identification was performed using 1% precursor and protein q-value. Quantification was based on the peak areas of extracted ion chromatograms (XICs) of 3 - 6 MS2 fragment ions, specifically b- and y-ions, q-value sparse data filtering was applied, and iRT profiling was selected. Differential protein abundance analysis was performed using a paired t-test, and p-values were corrected for multiple testing, using the Storey method [29, 30]. Protein groups with at least two unique peptides, q-value < 0.01, and absolute Log2(fold-change) > 0.58 were considered significantly altered.

**Figure 1.**
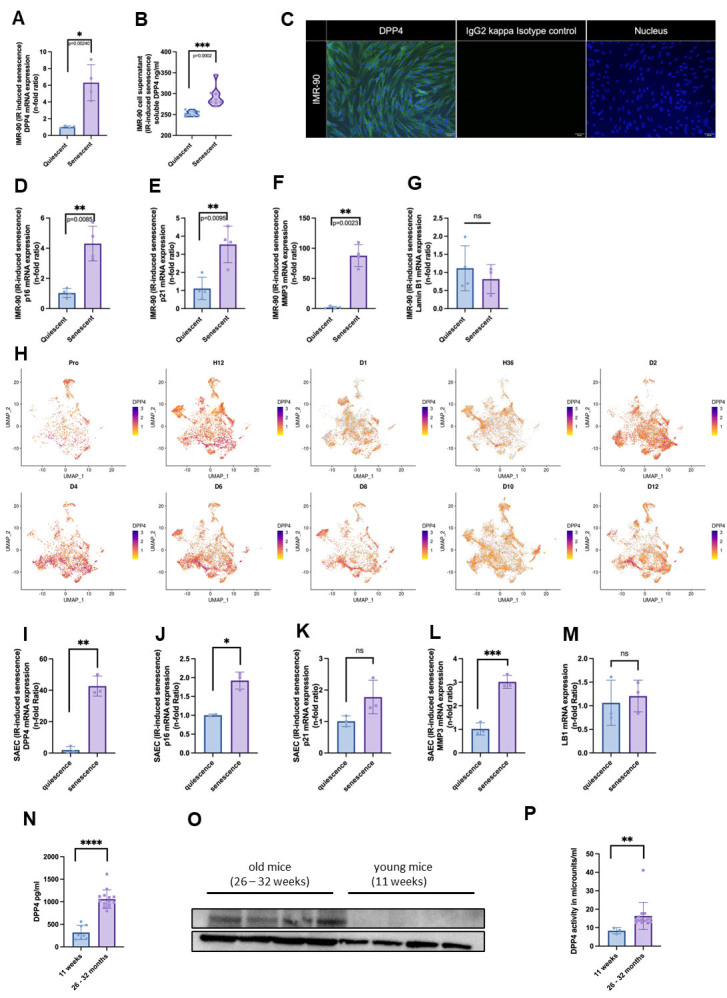
**DPP4 is upregulated in lung cells after induction of cellular senescence**. IMR-90 cells were induced to senescence, and (A) *DDP4* mRNA expression (n=4) and (B) soluble DPP4 in the supernatants of senescent cells compared to quiescent cells (n=8) were measured. (**C**) DPP4 is expressed in IMR-90, as detected by immunofluorescence staining, after the induction of senescence with irradiation (IR-induced senescence). Left: green DPP4; middle: IgG2 kappa Isotype control; right: nucleus stained with DAPI. (**D-F**) Senescence factors *p16*, *p21*, and *MMP3* were significantly upregulated, whereas (G) Lamin-B1 was downregulated in senescent cells compared to quiescent cells (n=4). (**H**) Based on single-cell sequencing, *DPP4* expression was determined 12 h, 24 h, 36 h, 2 d, 4 d, 6 d, 8 d, 10 d, and 12 d after senescence induction with doxorubicin (n=3). (**I**) In small airway epithelial cells, senescence was induced by IR and a significant upregulation of *DPP4* mRNA was detected, associated with (J) significant *p16*, (K) non-significant (ns) *p21*, (L) significant *MMP3*, and (M) ns *Lamin-B1* regulation in senescent cells compared to quiescent cells (n=3). (**N-P**) In murine model, young (11 weeks, n=8) and old (26-32 months, n=14) mice were used for bronchoalveolar lavage. (**N**) soluble DPP4 was significantly upregulated. (**O**) DPP4 of lung homogenization was detectable as upregulated in old mice using Western blotting. (**P**) DPP4 activity was significantly upregulated (11 weeks n=3, 26-32 months n=14). Statistical analysis was performed under the assumption of non-normal distribution with the Mann-Whitney U test (ns p > 0.05; * p ≤ 0.05; ** p ≤ 0.01; *** p ≤ 0.001; **** p ≤ 0.0001).

### Clustering Analysis

Partial least square-discriminant analysis (PLS-DA) of the proteomics data was performed using the package mixOmics in R (version 4.0.2) [31].

### Data Availability

Raw data and complete MS data sets have been uploaded to the Mass Spectrometry Interactive Virtual Environment (MassIVE) repository, developed by the Center for Computational Mass Spectrometry at the University of California San Diego, and can be downloaded using the following link: ftp://massive.ucsd.edu/MSV000092495 (log in as guest). In addition, the following information/ID number can access the full data set: MassIVE ID number: MSV000092495; ProteomeXchange ID: PDX043991. (https://massive.ucsd.edu/ProteoSAFe/dataset.jsp?task=cb3ae649816b415b876181a9e82fb4bc)

### Statistical analysis

Data are presented as the mean ± standard error of the mean (SEM). Statistical analyses were performed using the GraphPad Prism 9 software (GraphPad, San Diego, USA) with Mann-Whitney U test, One-way ANOVA, and Two-way ANOVA (ns p > 0.05; * p ≤ 0.05; ** p ≤ 0.01; *** p ≤ 0.001; **** p ≤ 0.0001). The representative figures, which were not quantified themselves, were randomly chosen to support our results.

## RESULTS

### DPP4 expression is increased upon senescence induction in various lung cell types

To determine the effects of cellular senescence induction on DPP4 expression in the lungs, we used human primary lung fibroblasts (IMR-90), and serum-starved quiescent cells as controls. Senescence was induced using UV irradiation, then confirmed by detecting the upregulation of *p16* and *p21* and downregulation of *Lamin B1*. Additionally, we detected increased *DPP4* mRNA expression levels and increased levels of soluble DPP4 protein (Fig. 1A, B). The immunofluorescence signal of DPP4 in IMR-90 cells indicated a distribution of proteins at the cell surface (Fig. 1C). Additionally, the senescence-associated markers *p16* and *p21*, as well as the SASP factor matrix metalloproteinase-3 (*MMP3*), were upregulated in senescent cells compared to quiescent cells, whereas *Lamin B1* expression was decreased; however, this decrease was not statistically significant (Fig. 1D-G). We also performed RNA sequencing at different time intervals (12 h to 12 d) after irradiation to investigate the time course of *DPP4* RNA expression, which revealed a steady increase in expression over time (Fig. 1H).

In addition to fibroblasts, human lungs are mainly composed of epithelial cells that are competent for air exchange. To investigate *DPP4* expression in the epithelium, we used human primary small alveolar epithelial cells (SAECs). After irradiation-induced senescence, we detected an increase in *DPP4* mRNA expression levels (Fig. 1I). The senescence-associated marker *p16* mRNA was also significantly upregulated (although *p21* mRNA was not); *MMP3* mRNA, encoding a SASP factor, was also upregulated in senescent cells compared to quiescent cells, but no significant difference was detected in *Lamin B1* mRNA expression levels (Fig. 1J-M).

Finally, we determined DPP4 protein expression, secretion, and activity in bronchoalveolar lavage (BAL) using *in vivo* murine models (Fig. 1N-P). We used young (11 weeks) and old (26-32 month) C57bl/6 mice. After euthanasia, the thorax was opened, and BAL was performed using 500 µL of pre-warmed DPBS. By measuring the concentration of soluble (s)DPP4 protein levels in the BAL (Fig. 1N), we were able to detect an increase in sDPP4 in old mice compared to young mice, which could be confirmed by Western blotting (Fig. 1O). By using DPP4 activity assays, an increase in DPP4 activity in the BAL of old mice was detectable (Fig. 1P).

**Figure 2.**
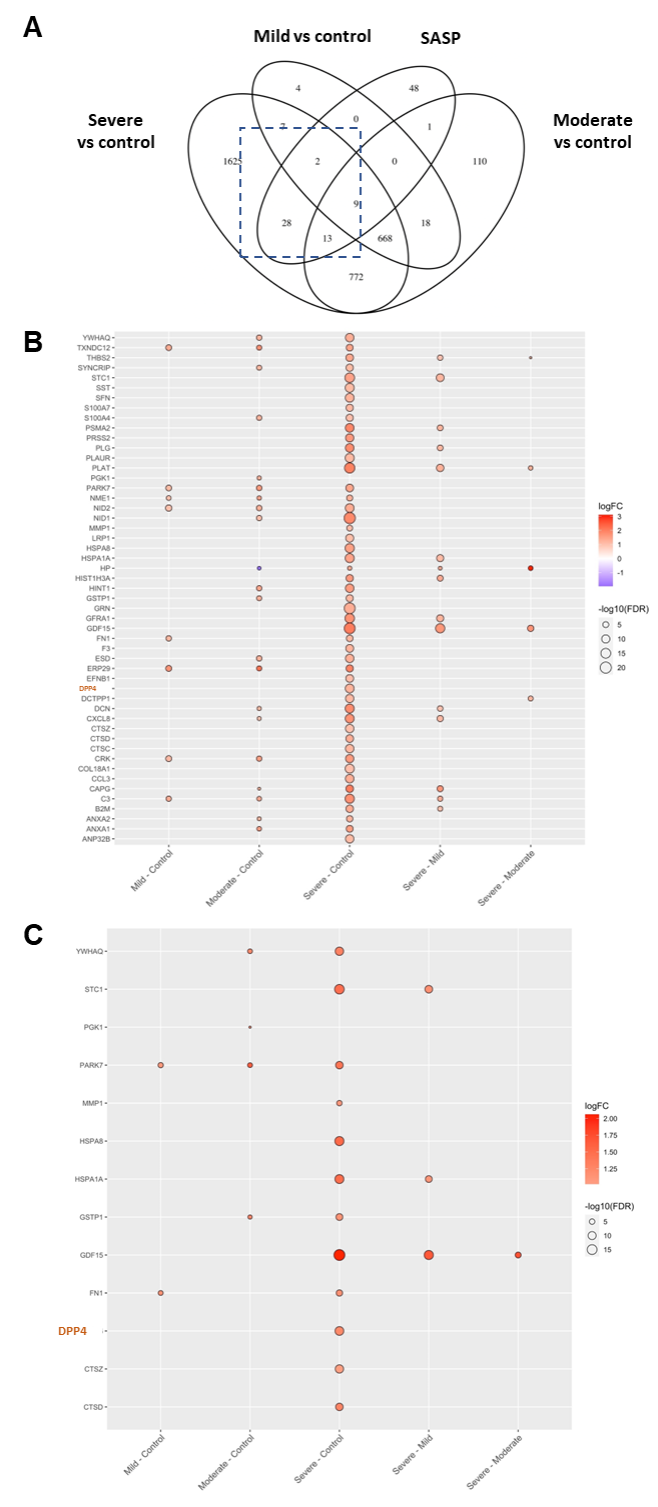
**Patients with a severe course of COVID-19 infection show higher expression of DPP4 compared to control**. (**A**) Venn-diagram showing age-matched proteins measured by SomaLogic protein assay using plasma from patients with mild (n = 35), moderate (n = 10), and severe (n = 42) COVID-19, as well as from controls (n = 33), and compared with the SASP identified from senescent IMR-90 cells. Differentially expressed genes were calculated using the R package DEseq2. p values were corrected for multiple comparison using Bonferroni correction. We selected a threshold of FDR < 0.05 and abs(logFC) > 1 for significance. Dashed box indicates further analyzed proteins. (**B**) Heatmap of 52 proteins of age-matched COVID-19 plasma samples with the SASP proteins of irradiated fibroblasts. (**C**) SASP proteins were highly upregulated: Dipeptidylpeptidase-4 (DPP4), Growth Differentiation Factor 15 (GDF15), Tyrosine 3-Monooxygenase/Tryptophan 5-Monooxygenase Activation Protein Theta (YWHAQ), Stanniocalcin 1 (STC1), Parkinsonism Associated Deglycase (PARK7), Matrix Metallopeptidase 1 (MMP1), Heat Shock Protein Family A (Hsp70) Member 8 (HSPA8), Heat Shock Protein Family A (Hsp70) Member 1A (HSPA1A), Glutathione S-Transferase Pi 1 (GSTP1), Fibronectin 1 (FN1), Cathepsin Z (CTSZ), and Cathepsin D (CTSD).

**Figure 3.**
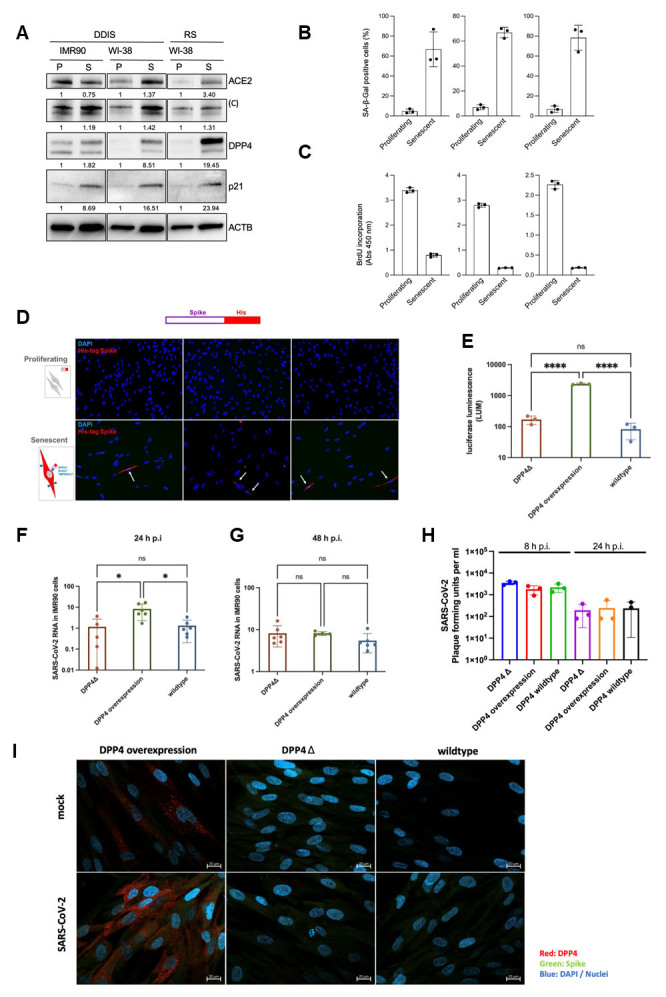
**DPP4 upregulation upon senescence induction with higher pseudotyped virus but not infectious particle upload**. (**A**) IMR-90 and Wi-38 fibroblasts were cultured at low population doubling levels (proliferative, P) or treated with etoposide to induce cellular senescence (S). DNA damage induced senescence (DDIS) was compared to replicative senescence (RS). Western Blotting for ACE2 and TMPRRS2 showed no difference in proliferative vs. senescent IMR-90 cells, but an increased signal in senescent Wi-38 cells was observed. DPP4 was upregulated in DDIS and RS Wi-38 cells, and p21 was increased in all conditions. (**B**) Senescence Associated-β-Galactosidase assays showed increased signals for all conditions (n=3). (**C**) BrdU incorporation was downregulated in senescent cells compared to proliferating cells (n=3). (**D**) Uptake of SARS-CoV-2 S (Spike) protein in proliferating vs. senescence cells. His-tagged SARS-CoV-2 Spike showed uptake in senescent cells (red signal), and nuclei stained in blue (DAPI). (**E**) Pseudoviral particles with a significantly higher luciferase luminescence signal in DPP4 overexpressing cells compared to knockout cells (DPP4Δ) and wild-type IMR-90 cells (n=3). (**F**) Significantly higher RNA detectable in SARS-CoV-2-infected IMR-90 cells after 24 h in DPP4 overexpressing cells compared to DPP4Δ and wild-type IMR-90 cells (n=6). (**G**) no differences after 48 h p.i. (n=6). (**H**) Detection of infectious viral particles (3 different SARS-CoV-2 isolates) using standard plaque assay to determine the plaque forming units per ml (PFU) revealed no differences between DPP4 overexpressing cells compared to DPP4Δ and wild-type IMR-90 cells after 8 h and 24 h p.i. (n=3). (**I**) Immunofluorescence staining showed no uptake of SARS-CoV-2 (red DPP4, green spike protein of SARS-CoV-2, blue nucleus (DAPI)). Statistical analysis was performed under the assumption of non-normal distribution with the Mann-Whitney U test (ns p > 0.05; * p ≤ 0.05; ** p ≤ 0.01; *** p ≤ 0.001; **** p ≤ 0.0001).

### DPP4 expression is elevated in plasma from COVID-19 patients with a severe course of infection

We investigated DPP4 levels in patients with COVID-19 using the SomaLogic protein assay (Fig. 2). We collected plasma from patients with mild (n = 35), moderate (n = 10), and severe (n = 42) courses of infection. We also analyzed plasma from healthy volunteers (n = 33). Our results showed that DPP4 was significantly elevated in patients with a severe course of COVID-19 infection compared to the control group (Fig. 2A, B).

We compared the proteins of age-matched COVID-19 plasma samples (Fig. 2C) and detected 13 elevated proteins, including DPP4, that were common between the SASP and the plasma of patients with severe COVID-19 compared to the control COVID-19 plasma samples. In addition to several pro-inflammatory proteins, DPP4 was one of 52 detected factors. Differentially expressed proteins were investigated using the R package DEseq2 and are presented in an age-matched manner. Furthermore, various other SASP proteins were highly upregulated, such as growth differentiation factor 15 (GDF15), tyrosine 3-monooxygenase/tryptophan 5-monooxygenase activation protein theta (YWHAQ), stanniocalcin 1 (STC1), parkinsonism associated deglycase (PARK7), matrix metallopeptidase 1 (MMP1), heat shock protein family A (Hsp70) member 8 (HSPA8), heat shock protein family A (Hsp70) member 1A (HSPA1A), glutathione S-transferase Pi 1 (GSTP1), fibronectin 1 (FN1), cathepsin Z (CTSZ), and cathepsin D (CTSD).

### DPP4 upregulation upon senescence induction leads to higher pseudotyped virus but not infectious particle upload

To determine the role of DPP4 during SARS-CoV-2 infection, we first investigated the expression of receptors, including angiotensin-converting enzyme 2 (ACE2) and transmembrane protease serine subtype 2 (TMPRSS2) during cellular senescence. Using western blotting, we detected an upregulation at the protein level for ACE2, TMPRSS2, and DPP4 (Fig. 3A), whereas cellular senescence was confirmed by SA-ß-Gal assay and BrdU incorporation (Fig. 3B, C). To identify the impact of this upregulation on SARS-CoV-2 infection, we used His-tagged spike proteins in culture and detected positive signals by immunofluorescence in senescent cells (Fig. 3D).

To investigate the role of DPP4, we overexpressed or reduced (knockout) DPP4 expression in IMR-90 fibroblasts using the CRISP/Cas9 system in a lentiviral vector that also contained the luciferase gene (Fig. 3E). Western blotting analysis was used to assess DPP4 expression levels and reverse transcription (RT) followed by quantitative (q)PCR analysis was used to measure *DPP4* mRNA levels. Wild-type (WT) cells normally produce low amounts of DPP4, whereas senescence induction leads to an increased expression of DPP4. DPP4-knockout cells therefore express approximately the same amount of DPP4 as control cells.

The impact of DPP4 modulation on SARS-CoV-2 infection was determined using both pseudoviral particles and SARS-CoV-2 viruses, where the pseudoviral particles corresponded to the spike glycoproteins. We incubated IMR-90 cells with pseudoviral particles for 24 h and luciferase activity was measured by quantification of the luminescence signal. The luminescence signal obtained from DDP4-overexpressing cells infected with pseudoviral particles was significantly upregulated compared to the signal obtained from DPP4-knockout cells or control cells (Fig. 3E). These findings suggest the entry of pseudoviral particles via the DPP4 receptor into the DPP4-overexpressing IMR-90 cells. Because pseudoviral particles lack important viral features, we infected DPP4-overexpressing IMR-90, DPP4-knockout IMR-90, and wild-type IMR-90 cells with full SARS-CoV-2 virus in a BSL-3 facility. The results showed a higher RNA uptake of SARS-CoV-2 in DDP4-overexpressing cells compared to the other cell populations 24 h after infection, but not after 48 h p.i. (Fig. 3F, G).

**Figure 4.**
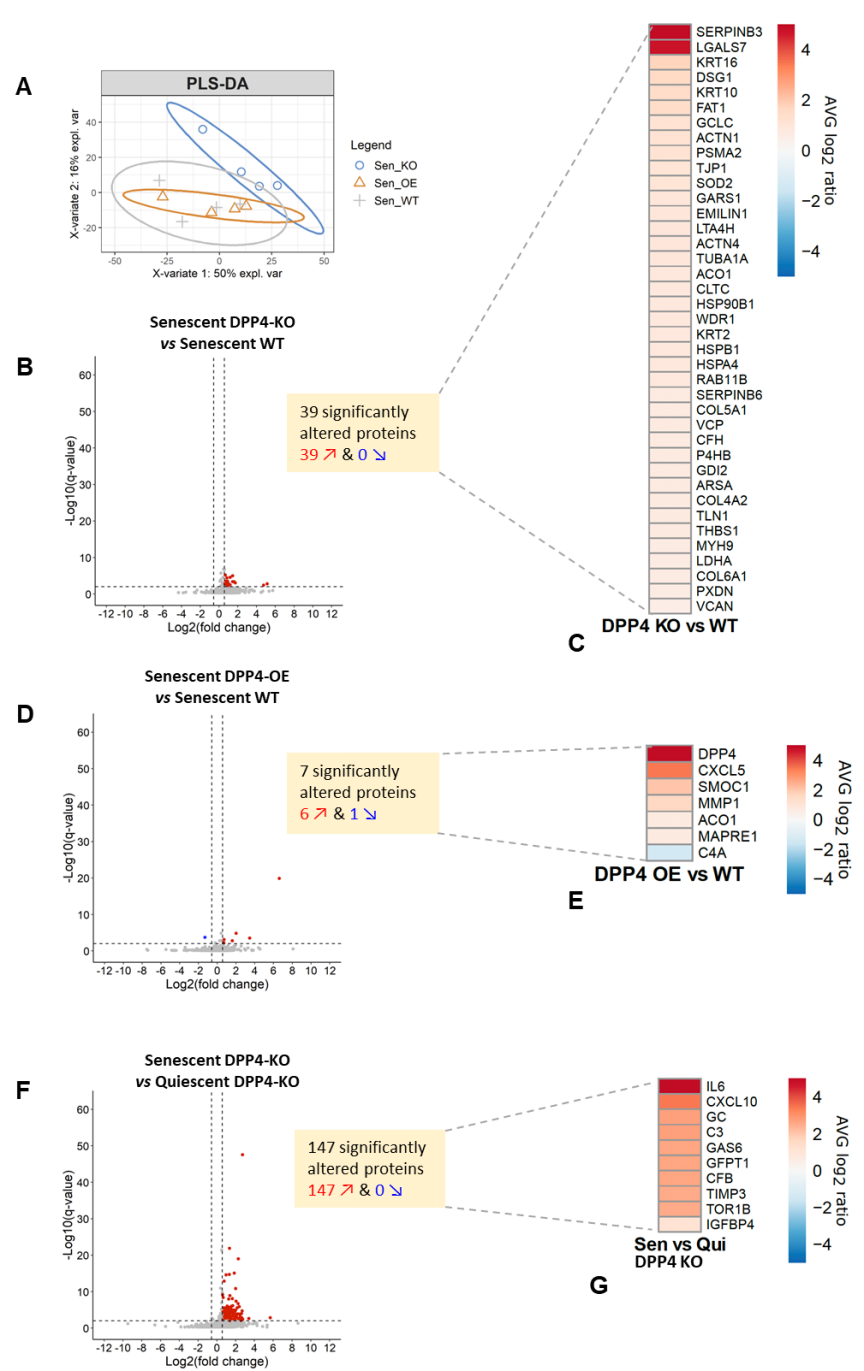
**Mass spectrometry analysis of IMR-90 cells with data-independent acquisition (DIA) on the Orbitrap Eclipse Tribrid platform**. (**A**) Supervised clustering using partial least square-discriminant analysis (PLS-DA) for IMR-90 wild-type (WT) cells, IMR-90 DPP4 overexpressing (OE) cells, IMR-90 DPP4 knockout (KO) cells, from both senescent and quiescent conditions. Senescent WT and DPP4-OE clustered together, but separately from senescent DPP4-KO, with 1,347 quantifiable protein groups. (**B**) Senescent DPP4-KO compared to senescent WT cells, with 39 significantly upregulated proteins. Protein Q-value < 0.01 & |Log2(FC)| > 0.58. (**C**) Heatmap showing upregulation of 39 proteins in senescent DPP4-KO compared to senescent WT cells. (**D**) Senescent DPP4-OE compared to senescent WT, with seven significantly altered proteins (six upregulated and one downregulated), Q-value < 0.01 & |Log2(FC)| > 0.58. (**E**) Heatmap showing upregulation of six proteins (including DPP4) and downregulation of complement factor C4A in senescent DPP4-OE compared to senescent WT cells. (**F**) Senescent DPP4-KO compared to quiescent DPP4-KO, with 147 significantly altered proteins (all upregulated). Q-value < 0.01 & |Log2(FC)| > 0.58. (**G**) Heatmap showing upregulation of ten proteins in senescent DPP4-KO compared to quiescent DPP4-KO cells.

To investigate the relevance of DPP4 in the replication of SARS-CoV-2, we performed plaque assays using viruses isolated from patients [20]. We used three different SARS-CoV-2 variants for infection (alpha variant, UK variant, and delta variant). To identify the effects on replication, we chose early (8 h) and late (24 h) timepoints after infection (Fig. 3H). The detection of infectious particles was carried out using plaque assays, and after 8 and 24 h no significant change was identified. These results were confirmed using immunofluorescence staining (Fig. 3I), i.e., no infection of IMR-90 cells by SARS-CoV-2 could be detected (no visible spike protein inside the cells).

### Identification of DPP4-regulated proinflammatory factors and tight junction proteins using mass spectrometry

The role of DPP4 as a SASP modulator during senescence induction was investigated using mass spectrometry (MS). We used quiescent, senescent wild-type (WT), senescent DPP4-overexpressing (OE), and senescent DPP4-knockout (KO) IMR-90 cells (Supplementary Fig. 2 and 3). After inducing senescence and quiescence, we collected conditioned media (CM) to perform an unbiased MS approach. The CM was then concentrated, proteolytically digested, and desalted. Measurements were performed using data-independent acquisition (DIA), a comprehensive quantitative proteomic approach as previously described for SASP analysis [6, 32, 33], on the Orbitrap Eclipse Tribrid platform (Thermo Fisher, CA, USA).

Partial least squares discriminant analysis demonstrated clustering of the individual groups, where the senescent DPP4-overexpressing cells were clustered with the wild-type senescent IMR-90 cells but were separated from senescent DPP4-KO cells (Fig. 4A). In total, 1,347 quantifiable protein groups were detected. To identify DPP4-related significantly upregulated proteins, we analyzed our data using volcano plots to differentiate between differentially upregulated and downregulated proteins. We identified 39 significantly altered proteins that were upregulated senescence-specific proteins in senescent DPP4-KO cells compared to senescent wild-type cells (Fig. 4B). We also detected upregulation of the tight junction protein zonula-occludens-1 (ZO-1, TJP1) in DPP4-KO cells (Supplementary Table 1, Fig. 4C). In addition, we observed an increase in proteins related to cell structure, including alpha-actin, myosin, thrombospondin, and protocadherin fat 1.

A comparison of senescent DPP4-overexpressing cells with wild-type senescent IMR-90 cells showed upregulation of six proteins (including DPP4) and downregulation of one protein (complement protein C4A) (Fig. 4D). The inflammatory chemokine CXCL5 and the major human interstitial collagenase MMP1 were upregulated (Fig. 4E). However, DPP4 knockout affected a large number of proteins in senescent and quiescent cells. In total, 147 significantly altered proteins were identified, with inflammatory active proteins (IL-6, CXCL10) being among the 10 most upregulated proteins (Fig. 4F, G).

### DPP4 regulates barrier function measured by transepithelial/transendothelial electrical resistance

To analyze the mechanistic impact of DPP4 on cell structure, we treated human primary corneal cells (CECs), which have a strong barrier function, with recombinant DPP4. CECs were treated with conditioned media (CM) from quiescent or senescent cells supplemented with a DPP4 inhibitor (sitagliptin) or recombinant DPP4 for up to six days. We then measured the transepithelial/ transendothelial electrical resistance (TEER) of the epithelial barrier to determine electrical resistance across the cellular monolayer under the influence of DPP4 or its inhibitor.

**Figure 5.**
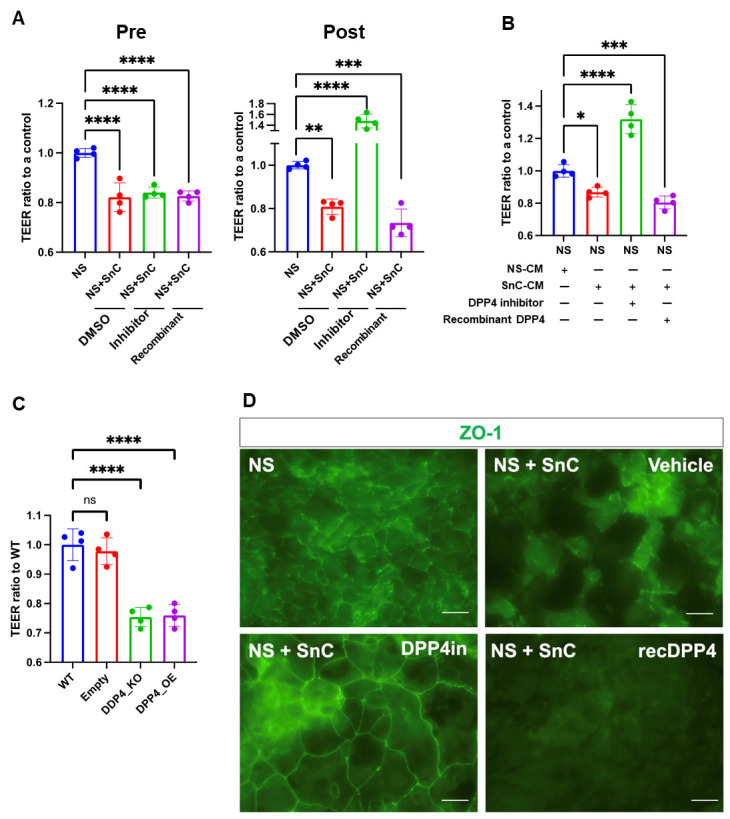
**DPP4 mediates epithelial barrier disruption**. (**A**) Transepithelial electrical resistance (TEER) in non-senescent (NS) and senescent corneal epithelial cells (SnC) was measured upon pre- and post-treatment with vehicle (DMSO), DPP4 inhibitor, or recombinant DPP4. (**B**) TEER performed post administration of conditioned media from NS and SnC treated with vehicle (DMSO), DPP4 inhibitor, or recombinant DPP4. (**C**) TEER in the different populations of corneal epithelial cells. WT: wild-type, OE: overexpression, KO: knockout. (**D**) Immunostaining of ZO-1 in NS/SnC cultures treated with DPP4 inhibitor, recombinant DPP4, or vehicle (DMSO). Scale bar indicates 50 µm. Results were plotted as the mean and standard deviation from four independent experiments, n=5. Statistical analysis was performed under the assumption of non-normal distribution with the Mann-Whitney U test (ns p > 0.05; * p ≤ 0.05; ** p ≤ 0.01; *** p ≤ 0.001; **** p ≤ 0.0001).

CECs rendered senescent (SnC) by the chemical agent doxorubicin were cultured along with non-senescent CECs (NS), and the epithelial barrier function was measured by calculating the TEER. SnC displayed an epithelial barrier function at a lower level than NS. Interestingly, modifying the levels of DPP4 impacted TEER ratios on day 6 (Post) (Fig. 5A). Treatment with the DPP4 inhibitor resulted in improved barrier resistance, suggesting that DPP4 impaired epithelial barrier function.

Next, we examined whether SASP factors could influence the epithelial barrier in the presence of DPP4 modulators. Interestingly, CM obtained from SnC disrupted the epithelial barrier, whereas the presence of the DPP4 inhibitor reversed this effect (Fig. 5B), suggesting that a lower expression of DPP4 protects against epithelial barrier disruption caused by the SASP. However, the genetically-modified DPP4 KO and OE IMR-90 cells both showed a reduced barrier function, suggesting that cells need to retain an appropriate level of DPP4 for barrier function (Fig. 5C).

Among the tight junction proteins, ZO-1 has an essential function in barrier integrity. As shown in the MS results, ZO-1 levels increased in DPP4-KO cells. In addition to performing TEER that allows a measurement of the overall barrier, we investigated the presence of ZO-1 by immunofluorescence 72 h after treating human primary corneal cells with recombinant DPP4 or DPP4 inhibitor (Fig. 5D). Here, senescent cells were cultivated with quiescent cells, identified by their typical larger morphology. In agreement with the data showing that DPP4 inhibitor significantly improved barrier function, ZO-1 immunofluorescence staining was stronger after treatment with the DPP4 inhibitor (Fig. 5D). In summary, we propose that DPP4 can disrupt the barrier of epithelial cells by reducing the levels of tight-junction protein ZO-1.

## DISCUSSION

Infectious diseases are associated with a significantly increased fatality rate in elderly patients [34]. In addition to increased comorbidities, specific aging factors also contribute significantly to higher morbidity and mortality rates. This association can likewise be observed in respiratory diseases, and notably in the ongoing COVID-19 pandemic, which has revealed a clear relationship between age and course of infection. However, the pathomechanisms that cause severe COVID-19 symptoms in the elderly have been poorly characterized at the molecular level.

Our study revealed that cellular senescence plays a major role in the pathogenesis of SARS-CoV-2 infection. Cellular senescence is characterized by an irreversible cell cycle arrest associated with the SASP [6]. Senescent cells create a pro-inflammatory environment upon the expression of myriad proteins [35]. The recently published human SASP-atlas provides insights into protein composition using a proteomic approach, highlighting DPP4 as one protein that is significantly increased upon senescence induction in human primary lung fibroblasts [6]. Interestingly, DPP4 is elevated at the cell surface in aged immune cells, such as monocytes and lymphocytes, and was reported to be a co-receptor for SARS-CoV in 2012 [36, 37].

In the present study, we used irradiation to trigger senescence in lung cells and performed validation studies by measuring the expression levels of *p16*, *p21*, and *MMP3*. Cellular senescence is considered a highly dynamic process, depending on the type of induction (e.g. irradiation vs. chemotherapeutic compound), but also depending upon the cell type, leading to a high variability of phenotype and SASP [38, 39]. Our results show an upregulation of *DPP4* in senescent cells compared to quiescent cells, both in culture and *in vivo*. Upregulation of *DPP4* has previously been reported in fibroblasts [7], and our results confirm these findings in lung cells.

Furthermore, as DPP4 functions as a cleavage protein and modulation of its enzymatic activity has been described as a regulatory mechanism [8], we investigated DPP4 activity in our study. We found that DPP4 activity increased in the supernatant of senescent cells, suggesting a potential role for soluble DPP4. In addition, RNA-seq data confirmed the increase in *DPP4* expression detected in IMR-90 cells following senescence induction.

To investigate the effects of COVID-19 infection on DPP4 levels, we used plasma samples from a cohort of patients with COVID-19. Our findings revealed age-dependent changes in protein distribution according to the severity of infection. We found that 13 SASP-related proteins, including DPP4, were increased in the cohort of elderly and severely ill COVID-19 patients.

Vankadari et al. [17] proposed that the S protein interacted with DPP4, and Li et al. [15] described the MERS receptor as a potential co-receptor for SARS-CoV-2. DPP4 is a highly active proteolytic protein involved in the cleavage of various substrates, such as glucagon-like-peptide [16]. The overall relevance of DPP4 as a protease for protein cleavage has been reported for several substrates [8]. However, the potential role of DPP4 in the cleavage of the spike protein of SARS-CoV-2 is still unclear. Recently published data have indicated various viral cleavage sites in the spike protein [18].

Our data indicate that DPP4 is not involved in the cell entry process of SARS-CoV-2. However, the increased expression of DPP4, as determined in senescent cells, may not only lead to a higher transmembrane-bound form of DPP4, but also to increased release of soluble DPP4. Soluble DPP4 may be able to cleave other proteins, including the spike glycoprotein, owing to its high proteolytic potential, and this cleavage could facilitate the viral entry of cells lacking proteases. Moreover, we determined that ACE2 and TMPRSS2 were also increased in senescent cells, in addition to DPP4.

SARS-CoV-2 RNA has been detected in various tissues, such as eye [40, 41] and brain [42], indicating viremia [43]. The presence of specific receptors for viral entry may explain these findings, and our data suggest that increased DPP4 expression, triggered upon senescence induction, may be involved in this phenomenon. However, it is unclear whether inhibition of DPP4 could improve the outcome of COVID-19 infection [44]. Inhibitors of viral entry must be taken early during viral infection to significantly modify the viral load; otherwise, the inhibitors will only achieve a reduction in symptoms, as for influenza virus therapy.

Interestingly, in agreement with our findings, Solerte et al. [45] described a reduced mortality in patients treated with DPP4 inhibitors. Additionally, Mirani et al. [46] reported an improvement in the clinical outcome of a small cohort of patients, and the possibility of targeting DPP4 as part of the senescence-associated genes for COVID-19 treatment was discussed by Maremande et al. [47].

Overall, the pathogenesis of respiratory viral infections should be considered in the context of age-related changes. Cellular senescence is a particularly promising subject of investigation because it creates an environment of pro-inflammation, which can lead to infections. We demonstrated here that some important viral receptors are overexpressed during cellular senescence, and although DPP4 does not have a high binding capacity to SARS-CoV-2, its proteolytic activity could still alter the structure of the virus to influence its cell entry.

To perform further mechanistic studies, we used lentiviruses to produce CRISP/Cas KO and overexpression constructs. We first infected these cells with a pseudotyped virus for 24 h and detected an increase in luciferase activity. Using SARS-CoV-2 in BSL-3 conditions, we also observed increased RNA levels in DPP4-overexpressing cells. To confirm this observation, we subsequently examined three different SARS-CoV-2 variants (alpha, beta, and delta) and determined viral replication by measuring the plaque forming units per ml. We did not detect any increased titers in the cells after 24 h. Our data indicate that there may be an initial advantage for the virus in DPP4-overexpressing cells. However, this does not affect the viral load during replication because expression of the S protein inside the cells could not be proven. Although our CRISP/Cas system was introduced into human primary cells, lung fibroblasts are not the site of SARS-CoV-2 replication.

To further investigate the effect of DPP4, we used the CM of DPP4-KO and DPP4-OE cells for MS analysis. We demonstrated that the pattern of protein expression in cells overexpressing DPP4 overlapped with those of senescent cells, confirming that DPP4 is expressed in senescence. In addition to pro-inflammatory factors that were upregulated in DPP4-KO cells, such as *IL-6* and *CXCL-6*, we demonstrated an effect on structure-forming proteins. ZO-1 is of particular interest because it is significantly involved in the barrier function of epithelial cells. We further investigated this mechanism through studies using human primary corneal cells. These cells have good barrier function and were tested in our study with recombinant DPP4 or with inhibitors. We showed that barrier function was significantly damaged by the targeted addition of DPP4, and that treatment with DPP4 inhibitors had a positive effect on the barrier, such as an increase in ZO-1 expression.

In conclusion, our findings indicate that DPP4 is a protein that affects the environment of senescent cells. DPP4 is present in both soluble and cellular forms, and we demonstrated that lung cells undergoing cellular senescence produced high levels of DPP4. We were also able to demonstrate a similar effect in mice by performing BAL *in vivo*.

The COVID-19 pandemic has generated substantial socioeconomic and economic damage. Our data show that DPP4 is significantly elevated in severely ill patients with COVID-19 in an age-matched comparison. We generated an *in vitro* cell model using CRISP/Cas, which is used to target infections with SARS-CoV-2. Although we were unable to demonstrate a direct effect of DPP4 on viral replication in lung fibroblasts, we did show the effect of the protein on the host. Moreover, using MS technology, we demonstrated a significant effect of DPP4 on the structural cellular components. ZO-1 was particularly affected, and our studies revealed that the DPP4 inhibitor had a positive effect on barrier function. Collectively, our studies demonstrate that DPP4, a specific factor upregulated during cellular senescence, can influence barrier function and respiratory infections.

## Supplementary Materials

The Supplementary data can be found online at: www.aginganddisease.org/EN/10.14336/AD.2023.0812.



## References

[1] López-Otín C, Blasco MA, Partridge L, Serrano M, Kroemer G (2013). The hallmarks of aging. Cell, 153:1194-1217. 23746838 10.1016/j.cell.2013.05.039 PMC3836174

[2] Chambers CR, Ritchie S, Pereira BA, Timpson P (2021). Overcoming the senescence-associated secretory phenotype (SASP): a complex mechanism of resistance in the treatment of cancer. Mol Oncol, 15:3242-3255. 34137158 10.1002/1878-0261.13042 PMC8637570

[3] Campisi J (1997). Aging and cancer: the double-edged sword of replicative senescence. J Am Geriatr Soc, 45:482-488. 9100719 10.1111/j.1532-5415.1997.tb05175.x

[4] Campisi J (2016). Cellular Senescence and Lung Function during Aging. Yin and Yang. Annals of the American Thoracic Society, 13 Suppl 5:S402-S406. 28005423 10.1513/AnnalsATS.201609-703AW PMC5291472

[5] Neri F, Basisty N, Desprez PY, Campisi J, Schilling B (2021). Quantitative Proteomic Analysis of the Senescence-Associated Secretory Phenotype by Data-Independent Acquisition. Current Protocols, 1:e32. 33524224 10.1002/cpz1.32 PMC7898702

[6] Basisty N, Kale A, Jeon OH, Kuehnemann C, Payne T, Rao C, et al. (2020). A proteomic atlas of senescence-associated secretomes for aging biomarker development. PLOS Biology, 18:e3000599. 31945054 10.1371/journal.pbio.3000599 PMC6964821

[7] Kim KM, Noh JH, Bodogai M, Martindale JL, Yang X, Indig FE, et al. (2017). Identification of senescent cell surface targetable protein DPP4. Genes & development, 31:1529-1534. 28877934 10.1101/gad.302570.117 PMC5630018

[8] Mulvihill EE, Drucker DJ (2014). Pharmacology, physiology, and mechanisms of action of dipeptidyl peptidase-4 inhibitors. Endocr Rev, 35:992-1019. 25216328 10.1210/er.2014-1035 PMC7108477

[9] Treskova-Schwarzbach M, Haas L, Reda S, Pilic A, Borodova A, Karimi K, et al. (2021). Pre-existing health conditions and severe COVID-19 outcomes: an umbrella review approach and meta-analysis of global evidence. BMC Medicine, 19:212. 34446016 10.1186/s12916-021-02058-6 PMC8390115

[10] O’Driscoll M, Ribeiro Dos Santos G, Wang L, Cummings DAT, Azman AS, Paireau J, et al. (2021). Age-specific mortality and immunity patterns of SARS-CoV-2. Nature, 590:140-145. 33137809 10.1038/s41586-020-2918-0

[11] Huang Y, Yang C, Xu X-f, Xu W, Liu S-w (2020). Structural and functional properties of SARS-CoV-2 spike protein: potential antivirus drug development for COVID-19. Acta Pharmacologica Sinica, 41:1141-1149. 32747721 10.1038/s41401-020-0485-4 PMC7396720

[12] Bangaru S, Ozorowski G, Turner HL, Antanasijevic A, Huang D, Wang X, et al. (2020). Structural analysis of full-length SARS-CoV-2 spike protein from an advanced vaccine candidate. Science, 370:1089. 33082295 10.1126/science.abe1502 PMC7857404

[13] Shang J, Wan Y, Luo C, Ye G, Geng Q, Auerbach A, et al. (2020). Cell entry mechanisms of SARS-CoV-2. Proceedings of the National Academy of Sciences, 117:11727. 10.1073/pnas.2003138117 PMC7260975 32376634

[14] Wang N, Shi X, Jiang L, Zhang S, Wang D, Tong P, et al. (2013). Structure of MERS-CoV spike receptor-binding domain complexed with human receptor DPP4. Cell Research, 23:986-993. 23835475 10.1038/cr.2013.92 PMC3731569

[15] Li Y, Zhang Z, Yang L, Lian X, Xie Y, Li S, et al. (2020). The MERS-CoV Receptor DPP4 as a Candidate Binding Target of the SARS-CoV-2 Spike. iScience, 23:101160. 32405622 10.1016/j.isci.2020.101160 PMC7219414

[16] Deacon CF (2019). Physiology and Pharmacology of DPP-4 in Glucose Homeostasis and the Treatment of Type 2 Diabetes. Frontiers in Endocrinology, 10. 30828317 10.3389/fendo.2019.00080 PMC6384237

[17] Vankadari N, Wilce JA (2020). Emerging WuHan (COVID-19) coronavirus: glycan shield and structure prediction of spike glycoprotein and its interaction with human CD26. Emerg Microbes Infect, 9:601-604. 32178593 10.1080/22221751.2020.1739565 PMC7103712

[18] Meyer B, Chiaravalli J, Brownridge P, Bryne DP, Daly LA, Agou F, et al. (2020). Characterisation of protease activity during SARS-CoV-2 infection identifies novel viral cleavage sites and cellular targets for drug repurposing. bioRxiv:2020.2009.2016.297945.

[19] Kitazawa K, Hikichi T, Nakamura T, Mitsunaga K, Tanaka A, Nakamura M, et al. (2016). OVOL2 Maintains the Transcriptional Program of Human Corneal Epithelium by Suppressing Epithelial-to-Mesenchymal Transition. Cell Rep, 15:1359-1368. 27134177 10.1016/j.celrep.2016.04.020

[20] Deinhardt-Emmer S, Böttcher S, Häring C, Giebeler L, Henke A, Zell R, et al. (2021). SARS-CoV-2 causes severe epithelial inflammation and barrier dysfunction. J Virol, 95:e00110-21. 33637603 10.1128/JVI.00110-21 PMC8139673

[21] Syed AM, Taha TY, Tabata T, Chen IP, Ciling A, Khalid MM, et al. (2021). Rapid assessment of SARS-CoV-2-evolved variants using virus-like particles. Science, 374:1626-1632. 34735219 10.1126/science.abl6184 PMC9005165

[22] Demaria M, O'Leary MN, Chang J, Shao L, Liu S, Alimirah F, et al. (2017). Cellular Senescence Promotes Adverse Effects of Chemotherapy and Cancer Relapse. Cancer Discov, 7:165-176. 27979832 10.1158/2159-8290.CD-16-0241 PMC5296251

[23] Chang BD, Swift ME, Shen M, Fang J, Broude EV, Roninson IB (2002). Molecular determinants of terminal growth arrest induced in tumor cells by a chemotherapeutic agent. Proc Natl Acad Sci U S A, 99:389-394. 11752408 10.1073/pnas.012602599 PMC117570

[24] Kitazawa K, Kawasaki S, Shinomiya K, Aoi K, Matsuda A, Funaki T, et al. (2013). Establishment of a Human Corneal Epithelial Cell Line Lacking the Functional TACSTD2 Gene as an In Vitro Model for Gelatinous Drop-Like Dystrophy. Investigative Ophthalmology & Visual Science, 54:5701-5711. 23868985 10.1167/iovs.12-11043

[25] Stuart T, Butler A, Hoffman P, Hafemeister C, Papalexi E, Mauck WM 3rd, et al. (2019). Comprehensive Integration of Single-Cell Data. Cell, 177:1888-1902.e1821. 31178118 10.1016/j.cell.2019.05.031 PMC6687398

[26] Anerillas C, Herman AB, Munk R, Garrido A, Lam KG, Payea MJ, et al. (2022). A BDNF-TrkB autocrine loop enhances senescent cell viability. Nat Commun, 13:6228. 36266274 10.1038/s41467-022-33709-8 PMC9585019

[27] Escher C, Reiter L, MacLean B, Ossola R, Herzog F, Chilton J, et al. (2012). Using iRT, a normalized retention time for more targeted measurement of peptides. Proteomics, 12:1111-1121. 22577012 10.1002/pmic.201100463 PMC3918884

[28] Bruderer R, Bernhardt OM, Gandhi T, Xuan Y, Sondermann J, Schmidt M, et al. (2017). Optimization of Experimental Parameters in Data-Independent Mass Spectrometry Significantly Increases Depth and Reproducibility of Results. Mol Cell Proteomics, 16:2296-2309. 29070702 10.1074/mcp.RA117.000314 PMC5724188

[29] Storey JD (2002). A direct approach to false discovery rates. Journal of the Royal Statistical Society: Series B (Statistical Methodology), 64:479-498.

[30] Burger T (2018). Gentle Introduction to the Statistical Foundations of False Discovery Rate in Quantitative Proteomics. J Proteome Res, 17:12-22. 29067805 10.1021/acs.jproteome.7b00170

[31] Rohart F, Gautier B, Singh A, Lê Cao K-A (2017). mixOmics: An R package for ‘omics feature selection and multiple data integration. PLOS Computational Biology, 13:e1005752. 29099853 10.1371/journal.pcbi.1005752 PMC5687754

[32] Gillet LC, Navarro P, Tate S, Röst H, Selevsek N, Reiter L, et al. (2012). Targeted Data Extraction of the MS/MS Spectra Generated by Data-independent Acquisition: A New Concept for Consistent and Accurate Proteome Analysis*. Molecular & Cellular Proteomics, 11:O111.016717. 10.1074/mcp.O111.016717 PMC3433915 22261725

[33] Collins BC, Hunter CL, Liu Y, Schilling B, Rosenberger G, Bader SL, et al. (2017). Multi-laboratory assessment of reproducibility, qualitative and quantitative performance of SWATH-mass spectrometry. Nat Commun, 8:291. 28827567 10.1038/s41467-017-00249-5 PMC5566333

[34] Glynn JR, Moss PAH (2020). Systematic analysis of infectious disease outcomes by age shows lowest severity in school-age children. Scientific Data, 7:329. 33057040 10.1038/s41597-020-00668-y PMC7566589

[35] Coppé J-P, Desprez P-Y, Krtolica A, Campisi J (2010). The senescence-associated secretory phenotype: the dark side of tumor suppression. Annual review of pathology, 5:99-118. 10.1146/annurev-pathol-121808-102144 PMC4166495 20078217

[36] Kim KM, Noh JH, Bodogai M, Martindale JL, Yang X, Indig FE, et al. (2017). Identification of senescent cell surface targetable protein DPP4. Genes Dev, 31:1529-1534. 28877934 10.1101/gad.302570.117 PMC5630018

[37] Raj VS, Mou H, Smits SL, Dekkers DHW, Müller MA, Dijkman R, et al. (2013). Dipeptidyl peptidase 4 is a functional receptor for the emerging human coronavirus-EMC. Nature, 495:251-254. 23486063 10.1038/nature12005 PMC7095326

[38] Kumari R, Jat P (2021). Mechanisms of cellular senescence: cell cycle arrest and senescence associated secretory phenotype. Front Cell Dev Biol, 9:645593. 33855023 10.3389/fcell.2021.645593 PMC8039141

[39] Di Micco R, Krizhanovsky V, Baker D, d’Adda di Fagagna F (2021). Cellular senescence in ageing: from mechanisms to therapeutic opportunities. Nature Reviews Molecular Cell Biology, 22:75-95. 33328614 10.1038/s41580-020-00314-w PMC8344376

[40] Armstrong L, Collin J, Mostafa I, Queen R, Figueiredo FC, Lako M (2021). In the eye of the storm: SARS-CoV-2 infection and replication at the ocular surface? Stem cells Transl Med, 10(7):976-986 33710758 10.1002/sctm.20-0543 PMC8235146

[41] Kitazawa K, Deinhardt-Emmer S, Inomata T, Deshpande S, Sotozono C (2021). The Transmission of SARS-CoV-2 Infection on the Ocular Surface and Prevention Strategies. Cells, 10:769. 33918318 10.3390/cells10040796 PMC8065845

[42] Hansel C, Jendrossek V, Klein D (2020). Cellular Senescence in the Lung: The Central Role of Senescent Epithelial Cells. International journal of molecular sciences, 21:3279. 32384619 10.3390/ijms21093279 PMC7247355

[43] Deinhardt-Emmer S, Wittschieber D, Sanft J, Kleemann S, Elschner S, Haupt KF, et al. (2021). Early postmortem mapping of SARS-CoV-2 RNA in patients with COVID-19 and the correlation with tissue damage. Elife, 10: e60361. 33781385 10.7554/eLife.60361 PMC8009677

[44] Pitocco D, Tartaglione L, Viti L, Di Leo M, Pontecorvi A, Caputo S (2020). SARS-CoV-2 and DPP4 inhibition: Is it time to pray for Janus Bifrons? Diabetes research and clinical practice, 163:108162-108162. 32335097 10.1016/j.diabres.2020.108162 PMC7179491

[45] Solerte SB, Di Sabatino A, Galli M, Fiorina P (2020). Dipeptidyl peptidase-4 (DPP4) inhibition in COVID-19. Acta Diabetol, 57:779-783. 32506195 10.1007/s00592-020-01539-z PMC7275134

[46] Mirani M, Favacchio G, Carrone F, Betella N, Biamonte E, Morenghi E, et al. (2020). Impact of Comorbidities and Glycemia at Admission and Dipeptidyl Peptidase 4 Inhibitors in Patients With Type 2 Diabetes With COVID-19: A Case Series From an Academic Hospital in Lombardy, Italy. Diabetes Care, 43:3042-3049. 33023989 10.2337/dc20-1340

[47] Maremanda KP, Sundar IK, Li D, Rahman I (2020). Age-Dependent Assessment of Genes Involved in Cellular Senescence, Telomere, and Mitochondrial Pathways in Human Lung Tissue of Smokers, COPD, and IPF: Associations With SARS-CoV-2 COVID-19 ACE2-TMPRSS2-Furin-DPP4 Axis. Front Pharmacol, 11: 584637. 33013423 10.3389/fphar.2020.584637 PMC7510459

