# Supplementary material for: Role of the Senescence-Associated Factor Dipeptidyl Peptidase 4 in the Pathogenesis of SARS-CoV-2 Infection
Source: Aging Dis. 2024 May 7;15(3):1398–415. doi: 10.14336/AD.2023.0812 (PMC11081172; doi:10.14336/AD.2023.0812)
Supplement: Supplementary file 1 [file AD-15-3-1398-s.pdf]

SUPPLEMENTARY DATA

Role of the Senescence-Associated Factor Dipeptidyl Peptidase 4 in the Pathogenesis of SARS-CoV-2 Infection

Stefanie Deinhardt-Emmer, Sharvari Deshpande, Koji Kitazawa, Allison B. Herman, Joanna Bons, Jacob P. Rose, Prasanna Ashok Kumar, Carlos Anerillas, Francesco Neri, Serban Ciotlos, Kevin Perez, Nilay Köse-Vogel, Antje Häder, Kotb Abdelmohsen, Bettina Löffler, Myriam Gorospe, Pierre-Yves Desprez, Simon Melov, David Furman, Birgit Schilling, Judith Campisi

© 2023. Deinhardt-Emmer S. et al. Published online at http://www.aginganddisease.org/EN/10.14336/AD.2023.0812

SUPPLEMENTARY DATA

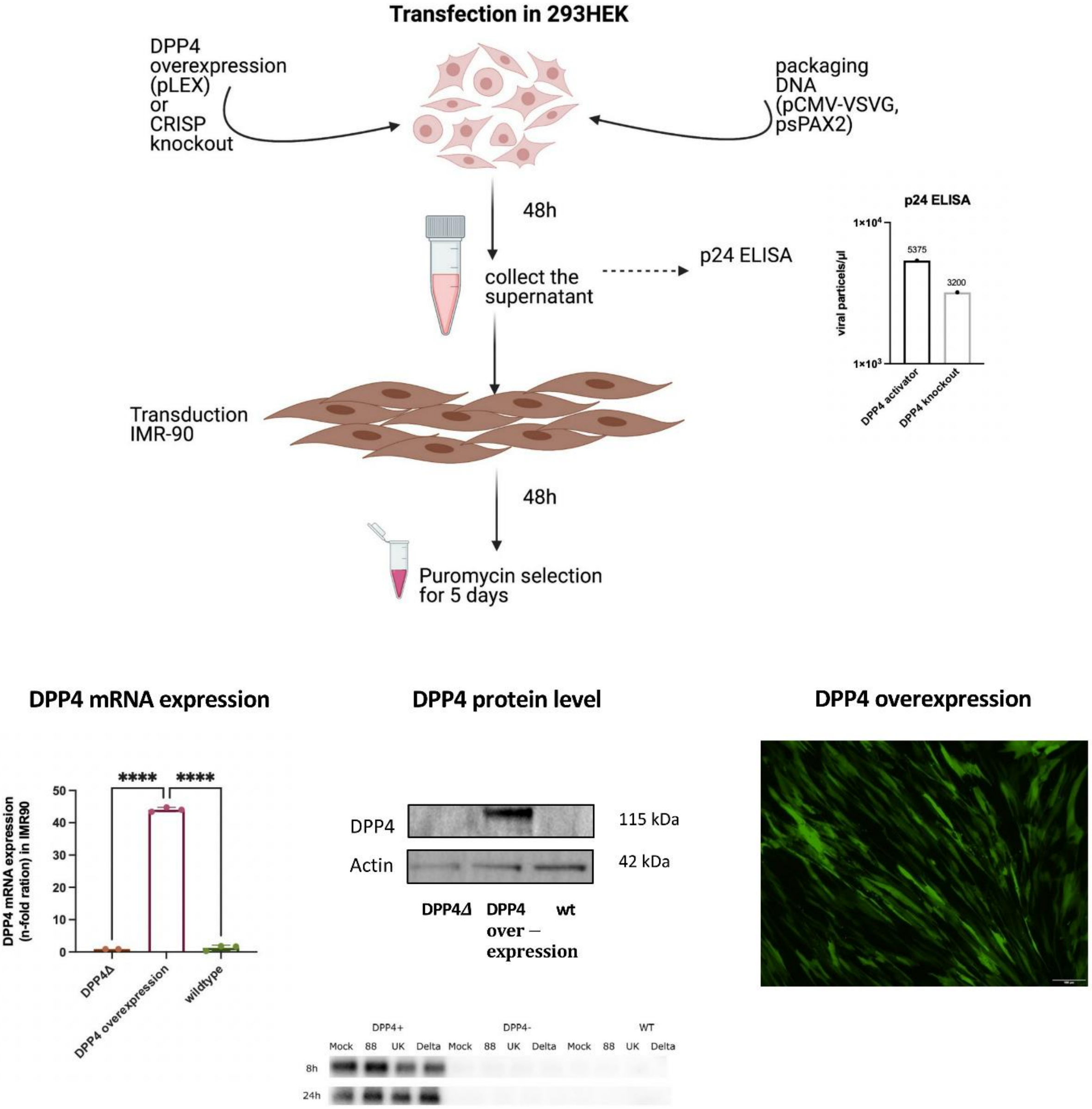

Supplementary Figure 1. Schematic of DPP4 CRISP/Cas9 overexpression and knockout. Shown is the transfection of 293HEK cells and puromycin selection, analysis of *DPP4* mRNA expression, DPP4 protein level using western blotting, and immunofluorescence.

© 2023. Deinhardt-Emmer S. et al. Published online at http://www.aginganddisease.org/EN/10.14336/AD.2023.0812

SUPPLEMENTARY DATA

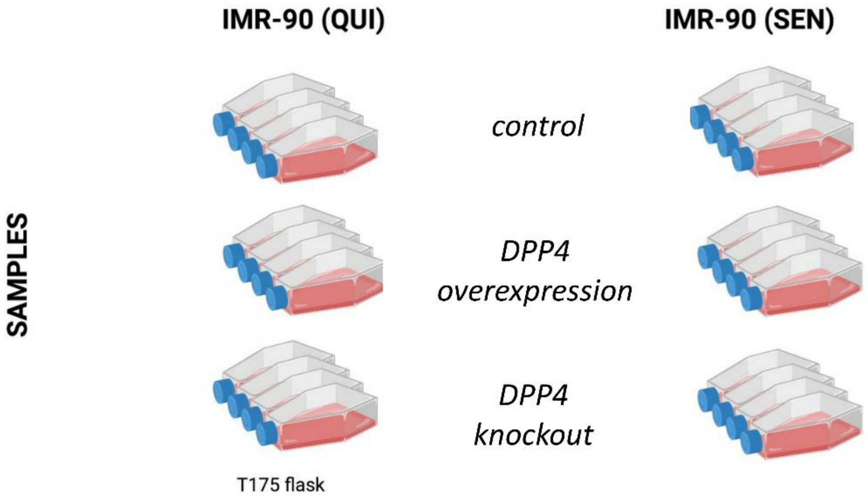

Supplementary Figure 2. **Experimental approach for mass spectrometry using quiescent and senescent IMR-90 cells.** For each group, 4 replicates of control cells, DPP4 overexpressing cells and DPP4 knockout cells were used.

© 2023. Deinhardt-Emmer S. et al. Published online at http://www.aginganddisease.org/EN/10.14336/AD.2023.0812

SUPPLEMENTARY DATA

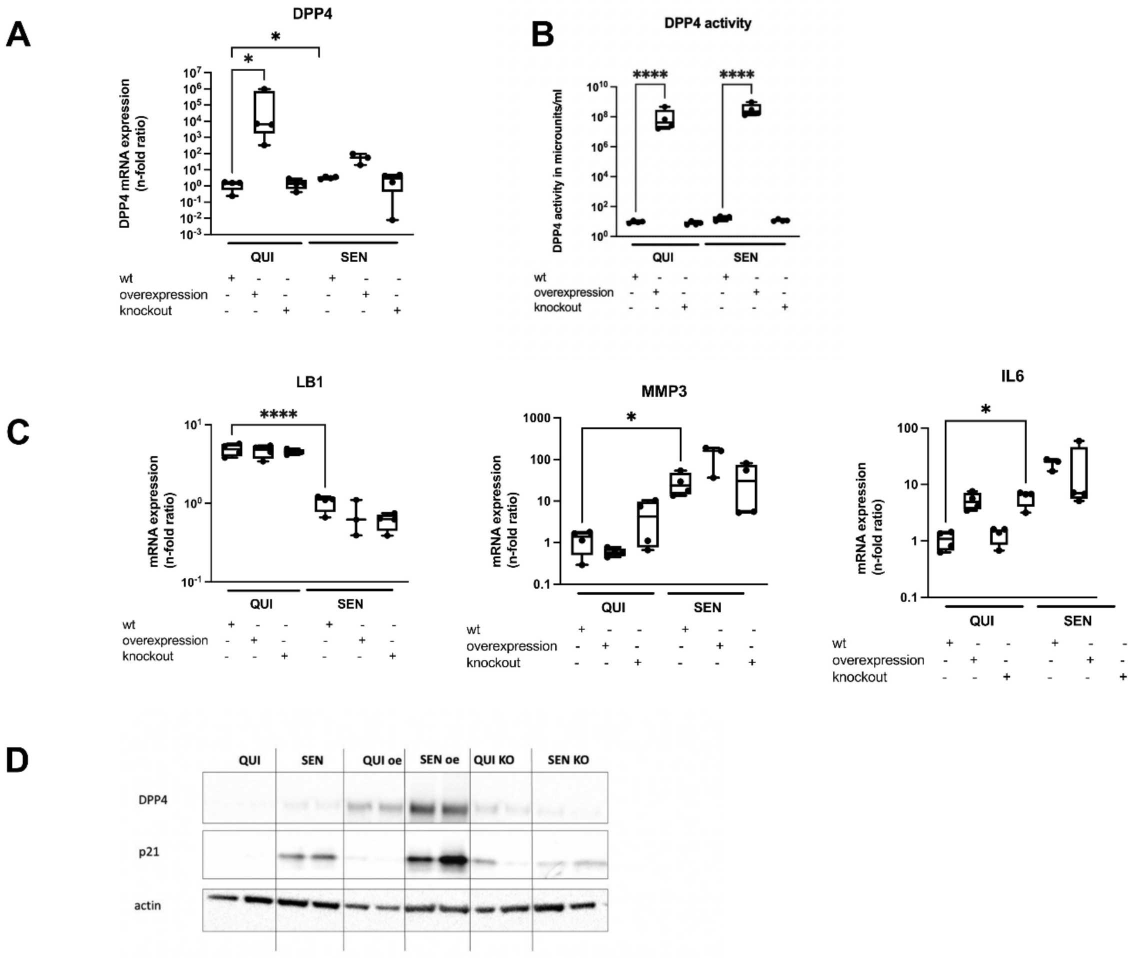

Supplementary Figure 3. **Validation of DPP4 knockout and overexpression.** (A) *DPP4* mRNA expression of quiescent cells compared to senescent cells, (B) DPP4 activity assay, (C) mRNA expression of the senescence markers *Lamin B1* (LB1), *matrixmetalloproteinase 3* (MMP3) and *interleukin 6* (IL6), (D) protein expression of p21 and DPP4 using western blotting.

© 2023. Deinhardt-Emmer S. et al. Published online at http://www.aginganddisease.org/EN/10.14336/AD.2023.0812
